# Editorial: Exercise and heart failure

**DOI:** 10.3389/fphys.2022.1030871

**Published:** 2022-09-16

**Authors:** Elisabetta Salvioni, Stefania Paolillo, Carlo Vignati, Damiano Magrì, Massimo Mapelli, Piergiuseppe Agostoni

**Affiliations:** ^1^ Centro Cardiologico Monzino, IRCCS, Milan, Italy; ^2^ Department of Advanced Biomedical Sciences, Federico II University of Naples, Naples, Italy; ^3^ Department of Clinical and Molecular Medicine, Azienda Ospedaliera Sant’Andrea, “Sapienza” Università Degli Studi di Roma, Rome, Italy; ^4^ Department of Clinical Sciences and Community Health, Cardiovascular Section, University of Milano, Milan, Italy

**Keywords:** heart failure, exercise, exercise physiology, oxygen uptake, quality of life

Heart failure (HF) is a complex disease that plagues public health with a still very high degree of mortality regardless of ejection fraction: reduced (HFrEF) or preserved (HFpEF) (Owan et al., 2006). The study of exercise physiology is of crucial importance to define the functional status of HF patients, to estimate prognosis, to guide therapy as well as to plan a possible rehabilitation program. In fact, exercise limitation is one of the cardinal symptoms of the patient with HF, not only in the case of maximal exertion, but also with regard to activities of daily living whose limitation negatively affects quality of life (Wilson and Mancini, 1993; Mapelli et al., 2020).

The driver of exercise is the metabolic cost coupled by the hemodynamic and respiratory response, so that the jamming of one of the “gears” participating in this complex mechanism is actually the main cause of the disease.

The most important parameter for measuring exercise capacity is oxygen uptake (VO_2_), which, according to Fick’s law, depends on cardiac output and arteriovenous oxygen content difference.

Therefore, at a first glance exercise limitation is basically due to insufficient or delayed supply of O_2_ to the muscle, and in particular to the mitochondria. The proper delivery of oxygen to exercising muscles mitochondria basically depends on three variables: O_2_ delivery, pO_2_ reduction from arteries to capillaries, and O_2_ diffusion from capillaries to the mitochondrion (Wagner, 1992; Wagner, 1996; Rovai et al., 2021).

**FIGURE 1:**
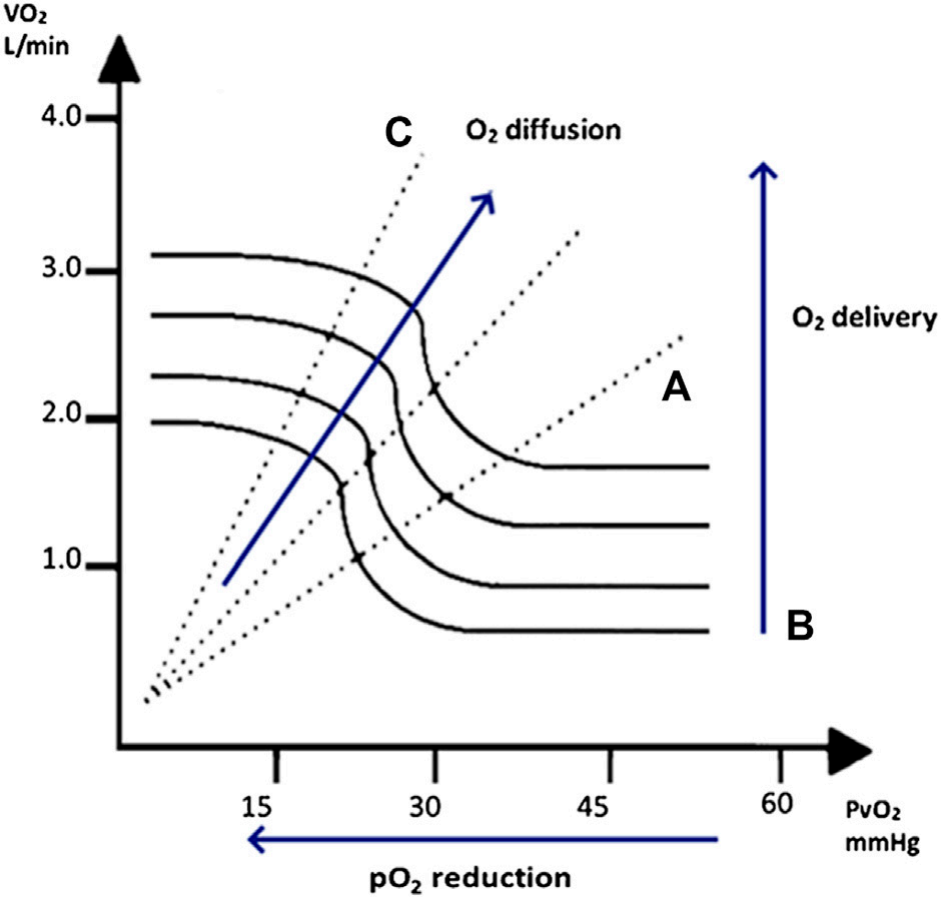
On the left, the relation between the O_2_ venous pressure (PvO_2_) and oxygen uptake (VO_2_) represented by Wagner diagram with the trend of O_2_ diffusion, O_2_ delivery, and O_2_ pression (pO_2_) reduction. During exercise VO_2_ increases by three independent mechanisms: **(A)** increase in O_2_ delivery (exercise-induced cardiac output increase and hemoconcentration (bold lines); **(B)** pO_2_ reduction in the capillary bed (*X* axis); and **(C)** differences in O_2_ diffusion from the capillary to the mitochondria (dashed lines). Reproduced with permission from Rovai et al., 2021 (Rovai et al., 2021).

The complex adjustment of these three factors is exemplified by Wagner’s diagram (Wagner, 1996; Rovai et al., 2021) (Figure 1), which shows:

-O_2_ delivery changes as a result of altered cardiac output, due to exercise induced cardiac output increase, redistribution of blood flow and hemoconcentration;
-pO_2_ reduction depending on O_2_ flow from the capillary to mitochondria which on its turn depends on the pO_2_ differences between capillaries and mitochondria and resistance to O_2_ flow;
-O_2_ diffusion from capillaries to the mitochondrion, which depends on length (distance from capillary to the mitochondria) and resistance to O_2_ flow due to fibrosis, cells, and whatever is located between capillaries and mitochondria.

The complexity of this system and the interdependence among the factors involved make the study of exercise physiology in the decompensated patient a difficult and heterogeneous task.

The articles collected in this Research Topic address this issue from different points of view.

Aerobic training is commonly used to improve patients exercise capacity. The randomized trial proposed by Gasser et al., will explore the field of exercise pathophysiology in HFpEF patients, whose diagnosis is still difficult and the underlying mechanisms not fully elucidated. As a consequence, also the rehabilitation programs need to be compared in an objective manner in this context, considering the pathophysiological mechanisms involved and their effectiveness. Specifically, the study aims to compare high-intensity interval training vs. moderate continuous training and it will focus not only on exercise capacity, but also on disease-specific blood biomarkers, cardiac and arterial vessel structure and function, total hemoglobin mass, metabolic requirements, habitual physical activity, and quality of life.

The paper by Paneroni et al. also fits into the landscape of studies of rehabilitation programs in patients with chronic HF. Here the authors proposed a novel approach to evaluate program effectiveness, using a battery of standardized activities of daily living, evaluated before and after the rehabilitation program. This strategy is particularly interesting because it evaluates performance based on activities that the patients perform in their normal life. Therefore, improvement is measured on activities well-known by the patient and most effectively reflects changes in their quality of life, mirroring exercises from everyday. From a physiological point of view cardiac rehabilitation involves all the three components of the Wagner diagram.

Differently, Wagner et al. work is based on the complex study of exercise physiology in a ramp protocol cardiopulmonary test, thus using a kind of exercise that is far away from every day activities, but, on the other hand, is highly controlled. Specifically, it focuses on the analysis of physiology, delving into the more complex mechanisms involved in the kinetics of exercise used as a mirror reflecting the efficiency of the system (i.e., the organism) in performing the effort. The authors found particularly meaningful the VO_2_ kinetic from the end of exercise to recovery transition, following ramp test termination (off-kinetics), a parameter that can be used as an alternative to peak VO_2_ when it is not reliable. We have no definite data to report on the original Wagner diagram (Figure 1) the time related changes of these variables involved in the VO_2_ kinetic of exercise recovery, but it is intriguing to speculate that O_2_ delivery reduction, i.e., mainly cardiac output and its distribution, is slowed down in HF patients compared to normal subjects, keeping VO_2_ reduction deferred.

The fourth paper by Aleksova et al. included in this Research Topic is a Review outlining the changes at the molecular level induced by exercise and the drugs available to modify these processes. Thus, in this case the study of HF exercise is done from a biomolecular perspective, analyzing the potentials expressed by different types of exercises, available programs, exercise-induced biological modifications, and additional complications due to concomitant conditions (e.g., vitamin D deficiency).

Taken together, the articles collected in this Research Topic show how broad the spectrum of analysis of HF and its relationship to exercise capacity is. Thus, there is no doubt that a great deal of work is still needed to investigate the different aspects, from the more molecular ones, to the physiology, to what can be considered a more “practical” approach. It is in fact of crucial importance to translate the application of the results obtained in the laboratory in the patient’s daily life, thus not stopping at the fundamental result of improving prognosis, but also focusing on everyday patients’ quality of life, considering the data obtained on the ability to perform efforts on a daily basis.

The evidence gathered draws attention to the importance of studying exercise as an expression of the patient’s ability to perform activity and thus a great indicator of their health and well-being, which is what really matters from a patient’s point of view. Moreover, the presented evidences further support the need to consider exercise as an indicator of the results achieved through drug therapy, as well as to consider physical activity a therapy itself.
